# nf-core/nanostring: a pipeline for reproducible NanoString nCounter analysis

**DOI:** 10.1093/bioinformatics/btae019

**Published:** 2024-01-11

**Authors:** Alexander Peltzer, Christopher Mohr, Kai B Stadermann, Matthias Zwick, Ramona Schmid

**Affiliations:** Clinical Bioinformatics and Systems Pharmacology, Translational Medicine and Clinical Pharmacology, Boehringer Ingelheim Pharma GmbH & Co. KG, 88400 Biberach/Riss, Germany; Clinical Bioinformatics and Systems Pharmacology, Translational Medicine and Clinical Pharmacology, Boehringer Ingelheim Pharma GmbH & Co. KG, 88400 Biberach/Riss, Germany; Clinical Bioinformatics and Systems Pharmacology, Translational Medicine and Clinical Pharmacology, Boehringer Ingelheim Pharma GmbH & Co. KG, 88400 Biberach/Riss, Germany; Clinical Bioinformatics and Systems Pharmacology, Translational Medicine and Clinical Pharmacology, Boehringer Ingelheim Pharma GmbH & Co. KG, 88400 Biberach/Riss, Germany; Clinical Bioinformatics and Systems Pharmacology, Translational Medicine and Clinical Pharmacology, Boehringer Ingelheim Pharma GmbH & Co. KG, 88400 Biberach/Riss, Germany

## Abstract

**Motivation:**

The NanoString™ nCounter^®^ technology platform is a widely used targeted quantification platform for the analysis of gene expression of up to ∼800 genes. Whereas the software tools by the manufacturer can perform the analysis in an interactive and GUI driven approach, there is no portable and user-friendly workflow available that can be used to perform reproducible analysis of multiple samples simultaneously in a scalable fashion on different computing infrastructures.

**Results:**

Here, we present the nf-core/nanostring open-source pipeline to perform a comprehensive analysis including quality control and additional features such as expression visualization, annotation with additional metadata and input creation for differential gene expression analysis. The workflow features an easy installation, comprehensive documentation, open-source code with the possibility for further extensions, a strong portability across multiple computing environments and detailed quality metrics reporting covering all parts of the pipeline. nf-core/nanostring has been implemented in the Nextflow workflow language and supports Docker, Singularity, Podman container technologies as well as Conda environments, enabling easy deployment on any Nextflow supported compatible system, including most widely used cloud computing environments such as Google GCP or Amazon AWS.

**Availability and implementation:**

The source code, documentation and installation instructions as well as results for continuous tests are freely available at https://github.com/nf-core/nanostring and https://nf-co.re/nanostring.

## 1 Introduction

The NanoString™ nCounter^®^ technology platform (Morrow and Donaldson 2011) is a widely used targeted quantification platform for the analysis of gene expression of up to ∼800 genes. The nCounter^®^ software suite nSolver™ provided by Nanostring Technologies can be used to perform data analysis, quality control and visualization although—as has been stated by other researchers already (Canouil *et al.* 2019)—the method only performs limited normalization and visualization. While these limitations can be partially overcome by utilizing other tools such as R package NACHO (Canouil *et al.* 2019), analyzing large numbers of samples can be cumbersome using a graphical user interface that requires dozens of settings to be configured manually and is thus not suitable for large-scale analysis projects, especially where automated reproducible analysis is key. To address these shortcomings, we developed a Nextflow (Tommaso *et al.* 2017)-based analysis pipeline within the nf-core (Ewels *et al.* 2020) framework. The pipeline can be used to perform QC, normalization, visualize optional output from nSolver^®^, annotate normalized gene expression tables in several standardized output formats. It also outputs a detailed report using MultiQC (Ewels *et al.* 2016), allowing users to assess the quality of their analyzed data quickly and explore some of the downstream analysis results that the pipeline automatically creates. Future developments will incorporate additional possibilities such as the analysis of GeoMx^®^ data and gene score computation within the pipeline.

## 2 Materials and methods

The nf-core/nanostring pipeline was written using Nextflow (Tommaso *et al.* 2017). Nextflow is a domain specific language that enables writing data intensive analysis pipelines in a reproducible, portable, and scalable way. We also utilized the community curated nf-core (Ewels *et al.* 2020) template for the development of the pipeline to follow best-practices from the community and adhere to well established standards. All individual parts of the pipeline are available as Docker and Singularity containers via the Biocontainers (Leprevost *et al.* 2017) initiative to ensure reproducibility of each step of the analysis.

**Figure 1.**
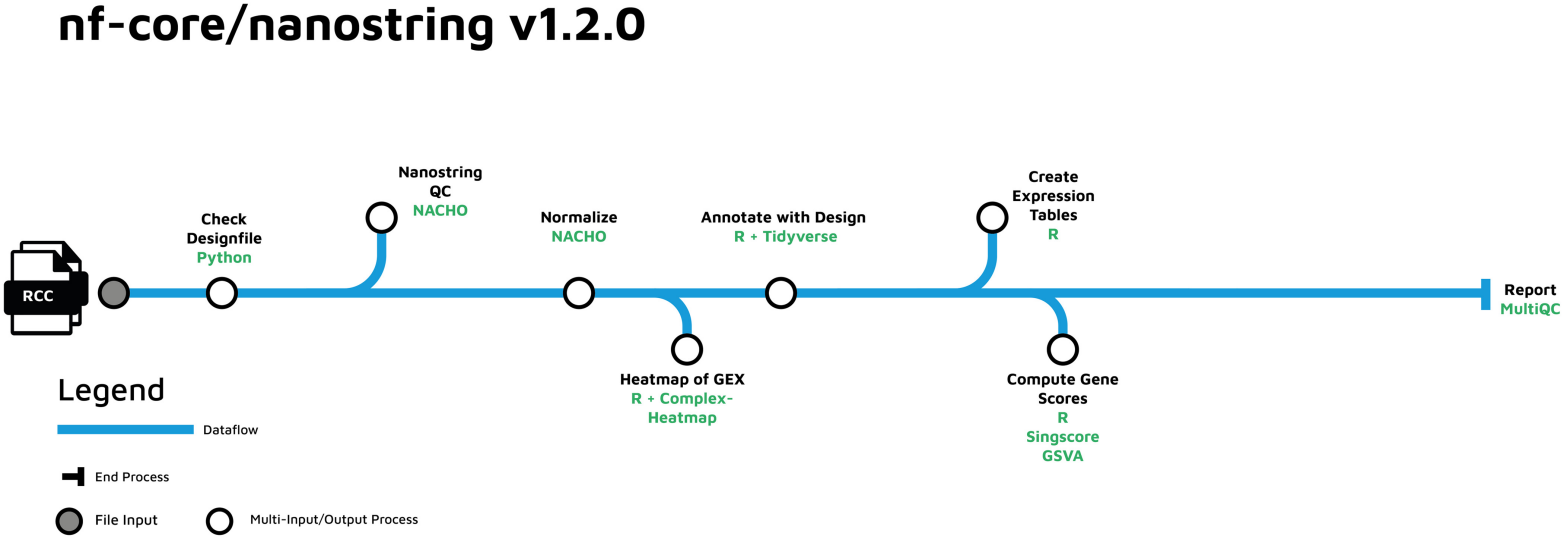
nf-core/nanostring pipeline outline, describing the individual pipeline steps.

The nf-core/nanostring pipeline comprised several components, that handle quality control of NanoString data, normalization of the obtained counts, annotation of the respective output matrix and the generation of expression files that allow for further downstream analyses. The outline of the pipeline is displayed in Fig. 1, whereas the individual steps are described further in the paragraph below.

## 3 Results

### 3.1 Design file

The pipeline requires a comma separated sample sheet file as input to describe samples and accompanying metadata. Mandatory input that needs to be specified is the location of RCC files as produced by the NanoString nCounter instrument and a sample identifier. Additional metadata can be provided by the user to allow for easier integration with downstream analysis methods.

Values can also be set to NA if not available and the pipeline will then e.g. exclude samples from such analysis where the respective metadata would be a requirement for further analysis.

The pipeline ultimately creates expression and normalized expression tables which can e.g. be used to perform differential gene expression analysis using other pipelines such as nf-core/differentialabundance (Manning *et al.* 2024) without a requirement to perform reformatting of the data tables.

### 3.2 Nanostring QC

This step of the pipeline performs quality control of the supplied RCC input data with the R package NACHO (Canouil *et al.* 2019) and thereby creates several diagnostic reports that can be used to assess sample quality with respect to key criteria as defined by the manufacturer. These QC metrics include Binding Density (BD), Field of View (FOV), Positive Control Linearity (PCL), Limit of Detection (LoD) as well as further key criteria required to assess the quality of a NanoString nCounter experiment. For a full list, please refer to the NACHO publication or GitHub page (https://github.com/mcanouil/NACHO) that explains the reports in more detail. The individual reporting images are furthermore integrated into the main pipeline report created via MultiQC to provide a comprehensive report for the entire pipeline run. This assists users to assess data quality and results jointly for all provided samples.

### 3.3 Normalize

The normalization step of the pipeline applies RCC normalization as described in the manufacturer documentation, e.g. takes both positive control and negative controls into account and can further be used to perform housekeeper normalization. Users can choose between both the default geometric mean (GEO) normalization (Nanostring Technologies Incorporated 2018) and the more advanced generalized linear model normalization (Wang *et al.* 2016) and can customize the normalization method to their liking. By utilizing the GEO normalization, the normalized data is equivalent to output of the Nanostring nCounter analysis software. Housekeeping gene-based normalization is used to correct for variability of sample input and therefore accounts for differences between lanes in terms of amount and quality of RNA (Nanostring Technologies Incorporated 2018). Users furthermore receive the raw counts, normalization factors, normalized counts in a standardized set of output files and can then thus check the effects of normalization on their respective data easily. Furthermore, users receive both housekeeping normalized and nonhousekeeping results automatically. The pipeline utilizes the implementation of above methods as integrated in the NACHO R (Canouil *et al.* 2019) package.

Future updates to the pipeline might include other options that have been shown to provide even improved normalization results in cases where technical replicates are available (Molania *et al.* 2019).

### 3.4 Annotation and expression table creation

Next, the normalized gene expression matrix is annotated by combining the design input about treatment, time, and other additional metadata with the normalized expression values to allow for further downstream analysis. Users can then load the tables in downstream methods and other analysis pipelines to e.g. perform differential gene expression analysis and/or pathway analysis.

### 3.5 Heatmap of gene expression

After normalization, the pipeline creates an overview heatmap (Gu *et al.* 2016) of gene expression values to assess gene expression intensities on a panel level. The heatmap is generated using standard R packages such as tidyverse (Wickham *et al.* 2019), ggplot2 (Wickham 2011), and ComplexHeatmap (Gu *et al.* 2016). This heatmap is also available in the final pipeline report.

### 3.6 Gene scores

Users can compute gene scores inside the pipeline by supplying a custom YAML formatted description file with the desired set(s) of genes and choosing one of the available methods for computing such gene scores. Available methods include PLAGE (Tomfohr *et al.* 2005), GSVA (Hänzelmann *et al.* 2013), SINGSCORE (Foroutan *et al.* 2021), SAMS (Souiai *et al.* 2001), and SSGSEA (Hänzelmann *et al.* 2013) as well as several other methodologies. Subsequently computed scores are then available in the final MultiQC report of the pipeline together with tables for further analysis.

### 3.7 Reporting

To facilitate an easy exploration and interpretation of a NanoString experiment results, the pipeline combines the output information of various steps to a comprehensive MultiQC HTML report. Individual results are combined in a general report table with accompanying visualizations [e.g. from the NanoString QC, Normalization and Heatmap (Gu *et al.* 2016) generation steps]. As such the report provides a one-stop shop to explore the outcome of any NanoString nCounter analysis, without having to investigate multiple output files for a basic assessment. This is further extended by more detailed reports such as the NACHO QC output report that enables a detailed assessment of the NanoString experiment.

### 3.8 Testing

The pipeline utilizes and benefits from the standardized nf-core approach of testing the pipeline automatically. The accompanying test data is available via the nf-core testing data GitHub repository (Nanostring Test Data) which is also referenced in the respective continuous testing profiles that check the pipeline for consistency (Nanostring Test Profiles). Furthermore, the nf-core website hosts the documentation and the full output of such automated testing runs of the pipeline, so that users can check prior running any tests themselves what the pipeline outputs look like: (nf-co.re/nanostring Results).

## 4 Discussion

The nf-core/nanostring pipeline has been built using Nextflow and the latest nf-core framework standards (nf-core tools 2.10) to enable both standardized, portable, and reproducible analyses of NanoString nCounter data. The native support for containerization technologies, direct integration into several cloud computing providers supported by Nextflow and several other technological advances such as the pipeline resume feature make the pipeline easy to use. This also holds true in the case of beginners with low to none experience with NanoString nCounter analysis. Additional advantages of the nf-core pipeline include live monitoring as well as standardized parameter documentation via the nf-core project (https://nf-co.re). The pipeline follows the latest nf-core framework template standards and thus supports external computing infrastructure configuration files that are easily adaptable via the nf-core community. Therefore, it can be used and deployed across various institutions and research facilities with very little effort.

The entire code base for the nf-core/nanostring workflow is available via GitHub in the main nf-core repository hosted for this pipeline [nf-core/nanostring: An analysis pipeline for Nanostring nCounter expression data. (github.com)]. Furthermore, all the documentation of the versioned pipeline is accessible via https://nf-co.re/nanostring and accompanied by automatically processed example datasets that users can browse at the following location permanently: https://nf-co.re/nanostring/results#nanostring. This facilitates uptake and understanding of data analysis results by the community and potential future users.

## 5 Conclusions

NanoString nCounter is a widely utilized method for the experimental analysis of up to 800 transcriptomic targets. The nSolver software provided by the manufacturer provides a feasible graphical workbench for the analysis of respective data, however the analysis of large-scale NanoString projects can be cumbersome. Especially when considering other important aspects of modern data analysis such as automation, portability and reproducibility of analyses, the available GUI-based methods cannot fulfill modern requirements. To facilitate the analysis of NanoString nCounter data, we developed an open-source, well documented, scalable, and reproducible Nextflow-based analysis pipeline. The nf-core/nanostring pipeline provides several improvements for researchers searching for a full analysis solution for NanoString nCounter data. The pipeline can handle thousands of samples simultaneously and perform many of the required steps for quality control, normalization, and standardized reporting for NanoString data. Future additions will provide further analysis possibilities such as the integration of improved normalization methods that can be leveraged in an automated fashion and might help to improve the usefulness of the pipeline even further. Here, the pipeline also benefits from the community-driven approach as a part of the nf-core community. Other future directions might involve the ability to perform more advanced differential gene expression analyses, potentially providing output for more complex analysis projects and visual exploration.
